# HPV16 E2 could act as down-regulator in cellular genes implicated in apoptosis, proliferation and cell differentiation

**DOI:** 10.1186/1743-422X-8-247

**Published:** 2011-05-20

**Authors:** Eric Ramírez-Salazar, Federico Centeno, Karen Nieto, Armando Valencia-Hernández, Mauricio Salcedo, Efraín Garrido

**Affiliations:** 1 Department of Genetics and Molecular Biology, CINVESTAV-IPN, Mexico City, Mexico; 2 Oncology Research Unit, Oncology Hospital, IMSS, Mexico City, Mexico; 3 National Institute of Genomic Medicine, SSA, Mexico City, Mexico; 4 Infection and Cancer Research Program, German Cancer Research Center, Heidelberg, Germany; 5 Mexican Institute of the Industrial Property, Mexico City, Mexico

## Abstract

**Background:**

Human Papillomavirus (HPV) E2 plays several important roles in the viral cycle, including the transcriptional regulation of the oncogenes E6 and E7, the regulation of the viral genome replication by its association with E1 helicase and participates in the viral genome segregation during mitosis by its association with the cellular protein Brd4. It has been shown that E2 protein can regulate negative or positively the activity of several cellular promoters, although the precise mechanism of this regulation is uncertain. In this work we constructed a recombinant adenoviral vector to overexpress HPV16 E2 and evaluated the global pattern of biological processes regulated by E2 using microarrays expression analysis.

**Results:**

The gene expression profile was strongly modified in cells expressing HPV16 E2, finding 1048 down-regulated genes, and 581 up-regulated. The main cellular pathway modified was WNT since we found 28 genes down-regulated and 15 up-regulated. Interestingly, this pathway is a convergence point for regulating the expression of genes involved in several cellular processes, including apoptosis, proliferation and cell differentiation; MYCN, JAG1 and MAPK13 genes were selected to validate by RT-qPCR the microarray data as these genes in an altered level of expression, modify very important cellular processes. Additionally, we found that a large number of genes from pathways such as PDGF, angiogenesis and cytokines and chemokines mediated inflammation, were also modified in their expression.

**Conclusions:**

Our results demonstrate that HPV16 E2 has regulatory effects on cellular gene expression in HPV negative cells, independent of the other HPV proteins, and the gene profile observed indicates that these effects could be mediated by interactions with cellular proteins. The cellular processes affected suggest that E2 expression leads to the cells in to a convenient environment for a replicative cycle of the virus.

## Background

Human Papillomavirus (HPV) is a small DNA virus that infects squamous epithelia performing a life cycle closely related to the differentiation program of the target cells [1]. HPV of the high-risk group (HR) as types 16 and 18, are associated with cervical cancer (CC) development while the low risk group as types 6 and 11, only with benign lesions. The HPV-HR E6 and E7 gene products are oncoproteins, since E6 binds to p53 inducing its degradation and blocking its function as tumor suppressor, while E7 binds proteins members of the "pocket" family as Rb, blocking its union to the transcription factor E2F and inducing the transcription of genes necessary for the transition towards S phase of the cell cycle [2]. The E6 and E7 gene expression is regulated in early stages of the viral infection by the E2 virus protein. This protein also plays several important roles in the viral cycle, since it regulates the replication of the viral genome together with E1 protein [3] and participates in the viral genome segregation through the cellular mitosis by its association with the cellular protein Brd4 [4]. During CC progression, the HPV genome is frequently integrated into cellular chromosomes loosing the expression of E2 and driving to an uncontrolled expression of E6 and E7, being this fact a critical step in cellular transformation [5-7]. Evidences indicate that E2 protein can regulate negative or positively the activity of several promoters of cellular genes, although the precise mechanism of this regulation is not yet well understood. For example, HPV-HR E2 protein negatively regulates the expression of β4-integrin gene [8], as well as the activity of the promoter of hTERT [9]. On the other hand, E2 has a positive regulation on the expression of several cellular genes including p21 [10], involucrin [11], and SF2/ASF [12] with an incomplete knowledge of the mechanism; however, it is believed that also involves its interaction with cellular proteins such as Sp1 [10], the transcription factor C/EBP [11], or TBP and components of the basal transcription machinery [12].

It has been demonstrated that the expression of E2 affects important cellular processes as cellular proliferation or death [13-15]. These effects are mainly mediated by its interaction with p53 [16,17] and possibly with TBP-associated factor 1 (TAF1), which regulates the expression of several genes that modulate cell cycle and apoptosis [18]. These interactions could induce changes in the expression of genes involved in these processes.

All the above mentioned reports have focused on analyzing the effects of E2 on particular promoters and very specific biological processes; therefore in this study our aim was to identify in a comprehensive way cellular genes and biological processes regulated by HPV16 E2. Using an adenoviral vector we expressed the HPV16 E2 gene in C-33A cells and analyzed the cellular gene expression profile generated by microarrays hybridization; ontological analysis indicated several pathways and cellular processes altered by HPV16 E2 expression.

## Methods

### Cell lines and culture conditions

The HEK293 and C-33A cell lines were obtained from ATCC. HEK293 cells were cultured in Dulbecco's modified Eagle's medium (DMEM), supplemented with 10% fetal bovine serum (FBS), penicillin (100 units/ml) and streptomycin (100 μg/ml). The C-33A cell line was cultured in Dulbecco's modified Eagle's medium: Nutrient Mixture F12 (DMEM-F12) supplemented with 10% Newborn Bovine Serum. Both cell lines were maintained in an humidified atmosphere at 37°C and 5% CO_2_.

### Recombinant Adenovirus and Infection

The construction of the replication deficient recombinant Adenovirus containing the E2 gene from HPV16 controlled by the cytomegalovirus promoter (CMV) was carried out with the Adeno-X Expression System (Clontech, Inc). The gene E2 was amplified by PCR using the primer forward 5'-TTCGGGATCCATGGAGACTCTTTGCCAACG-3' and the primer reverse 5'-ATCCGAATTCTCATATAGACATAAATCCAGTAG-3' using as a template the plasmid pcDNA3-E2. The corresponding amplicon was cloned in the pShuttle plasmid (Clontech, Inc) using the EcoRI and KpnI restriction sites. The generated pShuttle-E2 was digested with the restriction enzymes PI-SceI and I-CeuI to obtain the expression unit, and then clone it in the correspondent restriction sites of the pAdenoX plasmid (Clontech, Inc) generating the vector pAdenoX-E2. A pAdenoX-empty vector was also built, incorporating the PI-SceI-I-CeuI fragment from the pShuttle plasmid. This vector allowed us to generate an Adenovirus that does not contain expression cassette, denominated empty Adeno (Ad-empty). The recombinant viruses were generated by transfection into HEK293 cells with Lipofectamine Transfection Reagent (Invitrogen). The viral particles were propagated in HEK293 cells and purified using the system Adeno-X Mini Purification Kit (Clontech, Inc), following the instructions of the provider. The Adenovirus titer was obtained by immunocytochemistry following the protocol reported by Bewig [19]. For the infection of C-33A with the recombinant Adenoviruses, 800,000 cells were seeded in DMEM-F12 with 10% Newborn Bovine Serum and maintained at 37°C and 5% of CO_2 _during 24 hrs. The cell cultures were then incubated with 500 moi (multiplicity of infection) of either AdE2 HPV16 or Ad-Empty during 1.5 hrs in serum free DMEM-F12, in order to allow the virus adsorption. The viral stock was then removed away and the infection continued during 48 hrs in DMEM-F12 with 1.5% newborn bovine serum. To evaluate the infection efficiency, viral DNA was extracted using the Hirt method [20] and this material used as a template to amplify by PCR a 287 bp fragment of the Adenovirus 5 genome, using as a primer forward: 5'-TAAGCGACGGATGTGGCAAAAGTGA-3' and as a reverse 5'-CGTTATAGTTACGATGCTAGAGATT-3'.

### RT - PCR

Total RNA from the recombinant Adenovirus infected cells was obtained using the Trizol reagent (Invitrogen) following the indications of the provider. One μg of RNA was used to synthesize cDNA using M-MLV reverse transcriptase (Promega). This cDNA was used as a template to perform a PCR reaction using as primer forward 5'-TTCGGGATCCATGGAGACTCTTTGCCAACG-3' and as reverse 5'-ATCCGAATTCTCATATAGACATAAATCCAGTAG-3' amplifying a 1098 bp fragment corresponding to the full length HPV16 E2 gene. We used also a primer forward 5'-CTGTGGACCGTGAGGATA-3 and a reverse 5'-CTGTTGGGCATAGATTGTT-3' to amplify by PCR a 750 bp fragment of the Ad-5 Hexon gene.

### Hybridization and analysis of microarrays data

10 μg of total RNA were used for cDNA synthesis and labeling with SuperScript II kit (Invitrogen), using in a first array dUTP-Cy3 incorporation for Non-infected cells (N.I.) and dUTP-Cy5 for Ad-empty; and in a second array dUTP-Cy3 for Ad-empty and dUTP-Cy5 for AdE2; Fluorophorus incorporation efficiency was analyzed measuring absorbance at 555 nm for Cy3 and 655 nm for Cy5. Similar quantities of fluorophorus labeled cDNA were hybridized on the oligonucleotides collection 50-mer Human10K from MWGBiotech Oligo Bio Sets (Germany). Images of the microarrays were acquired and quantified in the scanner ScanArray 4000 using the QuantArray software from Packard BioChips (USA). A first analysis of the images and their data were performed using the Array-Pro Analyzer software from Media Cybernetics (USA). Data were then normalized and analyzed with genArise software (Institute of Cellular Physiology, UNAM) and genes with a Z score ≥ 1.8 or ≤ -1.8 were considered with altered expression. An ontological analysis was performed with selected data using PANTHER classification system (Protein ANalysis THrough Evolutionary Relationships).

### Quantitative Reverse Transcription PCR (RT-qPCR)

Total RNA from recombinant Adenovirus infected cells was obtained using Trizol reagent (Invitrogen) following the indications of the provider; 1 μg of RNA was used to synthesize cDNA with M-MLV reverse transcriptase (Promega) and this material used for relative quantification of the selected genes obtained from the microarrays analyses, by qPCR using the commercial kit ABSOLUTE qPCR SYBR Green Mix (Abgene) following the recommendations of the provider. The evaluation of the mRNA levels of the gene of constitutive expression GAPDH was used to normalize. Amplicons quantification was performed by double delta Ct (ΔΔCt) method. The primer sequences used were: for GAPDH as forward 5'-CATCTCTGCCCCCTCTGCTGA-3' and as reverse 5'-GGATGACCTTGCCCACAGCCT-3'; for N-MYC as forward 5'-TACCTCCGGAGAGGACACC-3' and as reverse 5'-CTTGGTGTTGGAGGAGGAAC-3'; for JAG1 as forward 5'-CTTCAACCTCAAGGCCAGC-3' and as reverse 5'-CTGTCAGGTTGAACGGTGTC-3'; and for MAPK13 as forward 5'-ATGTCTTCACCCCAGCCTC-3' and as reverse 5'TCCTCACTGAACTCCATCCC3'.

## Results

### E2 expression in AdE2 infected C-33A cells

**Figure 1:**
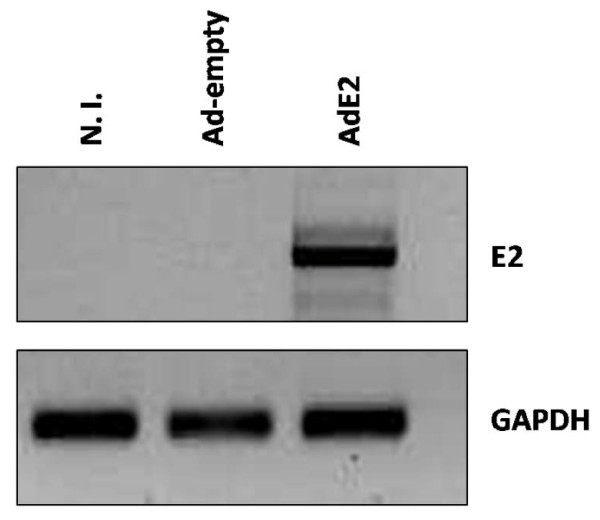
**Expression of E2 in AdE2 infected C-33A cells**. C-33A cells were infected with 500 moi of AdE2 or Ad-empty; 48 hours post-infection RNA was extracted from both cultures and also from non-infected cells (N.I.), cDNA was synthesized and PCR performed to detect the HPV16 E2 ORF. E2 was efficiently expressed in AdE2 infected C-33A cells; a comparison with GAPDH expression indicates the high E2 expression in those cells.

In order to obtain reliable data on the modifications induced by HPV16 E2 on the cellular gene expression profile, a recombinant adenoviral vector was used, since these vectors are able to efficiently infect cells from epithelial origin and the presence of a very strong promoter (MLP from HCMV) on its expression unit, guarantees a high efficiency of transgene expression. The infection of C-33A cells with the recombinant Adenovirus AdE2 was observed in almost 90% of the treated cell population (not shown). The evaluation of the E2 expression level in cells was carried out by RT-PCR 48 hours after infection, observing a very high amount of E2 mRNA in the infected cells (Figure 1).

### Differences in the transcriptional profiles

In order to identify the modifications induced on the cellular gene expression profile by HPV16 E2 gene, the expression profiles of AdE2 C-33A infected cells versus the expression profile of Ad-empty infected cells were obtained. To discriminate the effect that the adenoviral vector by itself had on the cellular gene expression, we also compared the expression profile of Ad-empty infected cells against that one from non-infected cells (mock). Data analysis obtained indicates that HPV16 E2 up-regulates 581 genes and down-regulates 1048 genes (Additional file 1, Table S1 and Additional file 2, Table S2). Table 1 shows a list of the 50 genes with higher induction (with a Z score >2.1) and Table 2 those 50 genes highly down-regulated (with a Z score <2.9) for the expression of E2. These results indicate that HPV16 E2 has preferably a negative effect on cellular gene expression.

**Table 1:** Highly up-regulated genes in C-33A cells by HPV16 E2 expression

Gene ID	Symbol	Name	Fold Change
NM_017839	LPCAT2	Lysophosphatidylcholine acyltransferase 2	5.3
NM_002071	GNAL	Guanine nucleotide binding protein (G protein)	4.9
NM_018667	SMPD3	Sphingomyelin phosphodiesterase 3	4.7
NM_004283	RAB3D	RAB3D, member RAS oncogene family	4.5
NM_006327	TIMM23	Translocase of inner mitochondrial membrane 23 homolog (yeast)	4.5
NM_017902	HIF1AN	Hypoxia inducible factor 1	4.2
NM_001421	ELF4	E74-like factor 4	3.9
NM_023919	TAS2R7	Taste receptor, type 2, member 7	3.9
NM_004289	NFE2L3	Nuclear factor (erythroid-derived 2)-like 3	3.9
NM_003630	PEX3	Peroxisomal biogenesis factor 3	3.8
NM_025087	CWH43	Cell wall biogenesis 43 C-terminal homolog (S. cerevisiae)	3.8
NM_013417	IARS	Isoleucyl-tRNA synthetase	3.8
NM_012338	TSPAN12	Tetraspanin 12	3.7
NM_004131	GZMB	Granzyme B (granzyme 2)	3.7
NM_002427	MMP13	Matrix metallopeptidase 13 (collagenase 3)	3.6
NM_017849	TMEM127	Transmembrane protein 127	3.6
NM_003821	RIPK2	Receptor-interacting serine-threonine kinase 2	3.6
NM_003958	RNF8	Ring finger protein 8	3.5
NM_004224	GPR50	G protein-coupled receptor 50	3.5
NM_001244	TNFSF8	Tumor necrosis factor (ligand) superfamily, member 8	3.4
NM_020375	C12orf5	Chromosome 12 open reading frame 5	3.4
NM_004219	PTTG1	Pituitary tumor-transforming 1	3.4
NM_005925	MEP1B	Meprin A, beta	3.4
NM_024730	RERGL	RERG/RAS-like	3.3
NM_014942	ANKRD6	Ankyrin repeat domain 6	3.3
NM_032946	NXF5	Nuclear RNA export factor 5	3.3
NM_004528	MGST3	Microsomal glutathione S-transferase 3	3.3
NM_012280	FTSJ1	FtsJ homolog 1 (E. coli)	3.3
NM_001372	DNAH9	Dynein, axonemal, heavy chain 9	3.2
NM_013256	ZNF180	Zinc finger protein 180	3.2
NM_003569	STX7	Syntaxin 7	3.2
NM_018370	DRAM1	DNA-damage regulated autophagy modulator 1	3.2
NM_017817	RAB20	RAB20, member RAS oncogene family	3.2
NM_020377	CYSLTR2	Cysteinyl leukotriene receptor 2	3.2
NM_022087	GALNT11	N-acetylgalactosaminyltransferase 11	3.2
NM_004426	PHC1	Polyhomeotic homolog 1 (Drosophila)	3.2
NM_031857	PCDHA9	Protocadherin alpha 9	3.2
NM_019863	F8	Coagulation factor VIII, procoagulant component	3.1
NM_018840	C20orf24	Chromosome 20 open reading frame 24	3.1
NM_006243	PPP2R5A	Protein phosphatase 2, regulatory subunit B', alpha isoform	3.1
NM_017946	FKBP14	FK506 binding protein 14, 22 kDa	3.1
NM_025212	CXXC4	CXXC finger 4	3.1
NM_012214	MGAT4A	Mannosyl (alpha-1,3-)-glycoprotein beta-1,4-N-acetylglucosaminyltransferase, isozyme A	3.1
NM_012087	GTF3C5	General transcription factor IIIC, polypeptide 5, 63 kDa	3.1
NM_017952	PTCD3	Pentatricopeptide repeat domain 3	3.1
NM_005967	NAB2	NGFI-A binding protein 2 (EGR1 binding protein 2)	3.1
NM_016021	UBE2J1	Ubiquitin-conjugating enzyme E2, J1 (UBC6 homolog, yeast)	3.1
NM_014945	ABLIM3	Actin binding LIM protein family, member 3	3.1
NM_001006	RPS3A	Ribosomal protein S3A	3.1
NM_012200	B3GAT3	Beta-1,3-glucuronyltransferase 3 (glucuronosyltransferase I)	3.1

Fold Change refers to differences in Z score values compared to expression in non-infected cells, analysis was perform using genArise software. Changes in gene expression with a cut-off >3.1 was used and the p-value was calculated by Student's t-test.

**Table 2:** Highly down-regulated genes in C-33A cells by HPV16 E2 expression

Gene ID	Symbol	Name	Fold Change
NM_001872	CPB2	Carboxypeptidase B2	-8.5
NM_024610	HSPBAP1	HSPB (heat shock 27 kDa) associated protein 1	-8.2
NM_022118	RBM26	RNA binding motif protein 26	-8.2
NM_017819	RG9MTD1	RNA (guanine-9-) methyltransferase domain containing 1	-8.1
NM_005319	HIST1H1C	histone cluster 1, H1c	-7.9
NM_000859	HMGCR	3-hydroxy-3-methylglutaryl-Coenzyme A reductase	-7.9
NM_003829	MPDZ	Multiple PDZ domain protein	-7.8
NM_017806	LIME1	Lck interacting transmembrane adaptor 1	-7.5
NM_005166	APLP1	Amyloid beta (A4) precursor-like protein 1	-7.5
NM_015985	ANGPT4	Angiopoietin 4	-7.3
NM_016283	TAF9	TAF9 RNA polymerase II, TATA box binding protein (TBP)-associated factor	-7.0
NM_000808	GABRA3	Gamma-aminobutyric acid (GABA) A receptor, alpha 3	-7.0
NM_005264	GFRA1	GDNF family receptor alpha 1	-7.0
NM_003420	ZNF35	Zinc finger protein 35	-7.0
NM_016179	TRPC4	Transient receptor potential cation channel, subfamily C, member 4	-6.9
NM_020200	PRTFDC1	Phosphoribosyl transferase domain containing 1	-6.9
NM_014736	KIAA0101	KIAA0101	-6.9
NM_020651	PELI1	Pellino homolog 1 (Drosophila)	-6.8
NM_006061	CRISP3	Cysteine-rich secretory protein 3	-6.8
NM_014626	TAAR2	Trace amine associated receptor 2	-6.8
NM_007048	BTN3A1	Butyrophilin, subfamily 3, member A1	-6.7
NM_007213	PRAF2	PRA1 domain family, member 2	-6.7
NM_024838	THNSL1	Threonine synthase-like 1 (S. cerevisiae)	-6.7
NM_012198	GCA	Grancalcin, EF-hand calcium binding protein	-6.6
NM_018938	PCDHB4	Protocadherin beta 4	-6.6
NM_006530	YEATS4	YEATS domain containing 4	-6.4
NM_000500	CYP21A2	Cytochrome P450, family 21, subfamily A, polypeptide 2	-6.4
NM_014547	TMOD3	Tropomodulin 3 (ubiquitous)	-6.4
NM_000228	LAMB3	Laminin, beta 3	-6.3
NM_002029	FPR1	Formyl peptide receptor 1	-6.3
NM_016508	CDKL3	Cyclin-dependent kinase-like 3	-6.3
NM_017779	DEPDC1	DEP domain containing 1	-6.3
NM_024612	DHX40	DEAH (Asp-Glu-Ala-His) box polypeptide 40	-6.3
NM_020666	CLK4	CDC-like kinase 4	-6.3
NM_007029	STMN2	Stathmin-like 2	-6.2
NM_012095	AP3M1	Adaptor-related protein complex 3, mu 1 subunit	-6.2
NM_018319	TDP1	Tyrosyl-DNA phosphodiesterase 1	-6.2
NM_024665	TBL1XR1	Transducin (beta)-like 1 X-linked receptor 1	-6.2
NM_012123	MTO1	Mitochondrial translation optimization 1 homolog (S. cerevisiae)	-6.2
NM_001285	CLCA1	Chloride channel accessory 1	-6.2
NM_025074	FRAS1	Fraser syndrome 1	-6.2
NM_017424	CECR1	Cat eye syndrome chromosome region, candidate 1	-6.1
NM_024756	MMRN2	Multimerin 2	-6.1
NM_002492	NDUFB5	NADH dehydrogenase (ubiquinone) 1 beta subcomplex, 5	-6.1
NM_004549	NDUFC2	NADH dehydrogenase (ubiquinone) 1, subcomplex unknown, 2	-6.1
NM_018128	TSR1	TSR1, 20S rRNA accumulation, homolog (S. cerevisiae)	-6.0
NM_000438	PAX3	Paired box 3	-6.0
NM_018991	STAG3L1	Stromal antigen 3-like 1	-6.0
NM_024576	OGFRL1	Opioid growth factor receptor-like 1	-6.0
NM_004249	RAB28	RAB28, member RAS oncogene family	-6.0

Fold Change refers to differences in Z score values compared to expression in non-infected cells, analysis was perform using genArise software. Changes in gene expression with a cut-off < -6.0 was used and the *p-*value was calculated by Student's t-test.

Gene Ontology analysis performed in PANTHER classification system (Protein ANalysis Through Evolutionary Relationships) [21] indicates that WNT pathway is the most severely affected by the expression of E2, since we found 28 genes down-regulated and 15 up-regulated. Interestingly, this pathway is a convergence point of genes involved in the regulation of several cellular processes, including apoptosis, cell proliferation and differentiation. We found modifications in the expression of genes that play an important role in regulating these processes, like the down-regulation of EGR2 and CASP9, both involved in apoptosis; the up-regulation of CCNA and down-regulation of RHOA, both involved in cell proliferation; and the down-regulation of some of the type I keratins, markers of epithelial cell differentiation such as keratin 14, 24 and 34. Moreover, we found that a large number of genes from pathways such as PDGF, angiogenesis and cytokines and chemokines mediated inflammation, are also altered for expression of HPV16 E2 (Tables 3 and 4).

**Table 3:** Top 10 up-regulated pathways in C-33A cells expressing E2.

Cellular Pathway	No. genes altered
**Wnt signaling pathway**	15
**Inflammation mediated by chemokine and cytokine signaling pathway**	10
**Angiogenesis**	8
**Integrin signalling pathway**	8
**Cadherin signaling pathway**	8
**B cell activation**	8
**Apoptosis signaling pathway**	7
**PDGF signaling pathway**	7
**EGF receptor signaling pathway**	7
**Oxidative stress Response**	6

Cellular Pathways with the greatest number of genes up-regulated by HPV16 E2 were selected as main pathways altered by E2. Ontological analysis was based on the algorithm used by PANTHER system (Protein ANalysis Through Evolutionary Relationships).

**Table 4:** Top 10 down-regulated pathways in C-33A cells expressing E2.

Cellular Pathway	No. genes altered
**Wnt signaling pathway**	28
**PDGF signaling pathway**	22
**Angiogenesis**	20
**Inflammation mediated by chemokine and cytokine signaling pathway**	20
**Integrin signalling pathway**	16
**TGF-beta signaling pathway**	13
**p53 pathway**	12
**FGF signaling pathway**	11
**Apoptosis signaling pathway**	10
**PI3 kinase pathway**	10

Cellular Pathways with the greatest number of genes down-regulated by HPV16 E2 were selected as main pathways altered by E2. Ontological analysis was based on the algorithm used by PANTHER system (Protein ANalysis Through Evolutionary Relationships).

### Validation of the microarrays data

**Figure 2:**
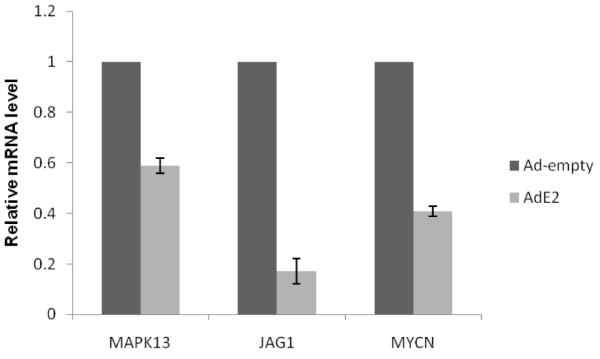
**Evaluation of mRNA levels of selected genes by RT-qPCR**. RNA from AdE2 and Ad-empty infected C-33A cells was extracted 48 hours post-infection, cDNA was synthesized, and specific primers used in a qPCR reaction to amplify fragments of the selected genes. Relative mRNA levels of MAPK13, JAG1 and MYCN genes are showed; values are expressed as difference in double delta-Ct compared with non-infected cells and expression of a housekeeping gene (GAPDH). Bars represent the mean ± SD (P < 0.05).

The validation of the results obtained with the microarrays analysis was performed by Real Time RT-qPCR, evaluating the mRNA levels of some of the genes negatively regulated by E2: MYCN, JAG1 and MAPK13. This approach was taking as basis recent results about validation of microarray experiments [22]. These genes were selected because they have a key role in some of pathways altered by HPV16 E2, such as apoptosis, cell cycle and keratinocyte differentiation [23-25]. Figure 2 shows the results of RT-qPCR for the selected genes among the AdE2 infected C-33A cells and those Ad-empty infected. The results of RT-qPCR from the selected genes correlate with the observations obtained with the microarrays analysis, indicating a very trusty landscape of the modifications on cellular gene expression induced by HPV16 E2.

## Discussion

In this work we report the modification of the gene expression profile induced by the expression of HPV16 E2.

We used the C-33A cell line to study the changes in the expression level of 10,000 human transcripts when the HPV16 E2 is expressed. In C-33A cells there is not evidence of HPV infection and they represent a convenient model to study the effect of E2 on cellular gene expression, without the involvement of another viral gene. Traditionally it has been considered that the effects observed in the regulation of cellular genes when the protein E2 is expressed in cervical carcinoma derived cell lines is due to the repression of the expression of the viral oncogenes E6 and E7 [2,26-28]; however, in this work we showed that HPV16 E2 induces changes in the expression of cellular genes, independently of the regulation of the viral oncoproteins E6 and E7.

The present study showed that HPV16 E2 importantly alters the expression profile of cellular genes, preferentially in a negative way, although a large number of genes were up-regulated.

It is well known that E2 protein suppress the activity of papillomavirus promoters by binding to low-affinity binding sites, leading to the displacement of cellular binding factors [8,29-31]. A similar scenario has been proposed for several cellular promoters, since in cultured primary keratinocytes it has been observed that HPV8 E2 represses the transcriptional activity of the β4 integrin promoter, due in part to its binding to a specific E2 binding site on the promoter and leading to displacement of at least one cellular DNA binding factor. However, growing evidence indicates that protein-protein interactions could be even more significant for E2-mediated transcriptional regulation of cellular genes since has been shown that E2 protein from several papillomavirus physically and functionally interact with a variety of cellular regulatory transcription factors, including Sp1, C/EBP, CBP/p300 and p53 [10,11,16,32,33]. The interaction with Sp1 is apparently one of the most relevant for transcriptional regulation of cellular genes by E2 since it is involved in the down-regulation of the hTERT promoter by HPV18 E2, but also the interaction with this transcription factor plays an important role in the transcriptional activation of several cellular promoters, including p21 by HPV18 E2 [10] or SF2/ASF by HPV16 E2 proteins [12].

Even when E2 shows cooperative activation with a variety of sequence-specific DNA binding factors such as AP1, USF, TEF-1, NF1/CTF, and C/EBP [11,34-37], a direct interaction between E2 and these cooperation partners has only been shown for HPV18 E2 with C/EBP, in the transactivation of the involucrin promoter [11], suggesting the transcriptional cooperation may also occur without a direct binding of these cellular proteins with E2.

The analysis of our data indicated that HPV16 E2 negatively regulates a higher number of genes (1048 genes) than those positively regulated (581 genes) (Additional file 1, Table S1 and Additional file 2, Table S2). In agreement with results previously reported [10,11], we found that HPV16 E2 up-regulate the expression of involucrin and cyclin-dependent kinase inhibitor 1A (p21) genes. However, we observed these genes up-regulated at a level below the established cutoffs for our analysis, indicating the relevance of the data set provided by our study for understanding the role of HPV E2 as a regulator of cellular gene expression.

Although we do not rule out the possibility that several genes are regulated by a direct interaction of E2 with specific sequences in the particular promoters, the global effect observed suggest that it could be the consequence of the interaction of E2 with several cellular transcription factors such as Sp1 (apparently the most important). E2 protein could destabilize protein-protein interactions between Sp1 and co-activators resulting in the negative regulation of the transactivation function of Sp1 itself, or E2 bound on the promoter via Sp1 may promote the recruitment of transcriptional co-activators such as p300/CBP and pCAF, leading to the transactivation of cellular promoters [38,39].

On the other hand, some HPV E2 proteins have been shown to interact with TBP and a number of components of the basal transcription machinery [18,40-45], regulating the recruitment of the pre-initiation complex and affecting both viral and cellular gene expression. Previous works in our group have demonstrated that E2 protein interacts and cooperates with TAF1 in the activation of E2-dependent viral promoters [18,40]. An analysis of our results indicates that 55 genes regulated by E2 have a natural regulation for TAF1 (data not shown).

Transregulation of specific cellular promoters could be also dependent on levels of the E2 protein in cells, since high levels of HPV16 E2 are known to result in inhibition of cell growth and promotion of apoptosis probably because with higher E2 levels, cellular metabolism may be compromised, leading to a reduced ability of cellular factors to control expression of several cellular genes. However, even we observed an abundant expression of E2, cell viability and different metabolic aspects of the cells (such as cell death) were not affected in a period of 72 hours post-infection with the AdenoE2 virus (data not shown).

This allows us to assume that the observed modulation of cellular gene expression by E2 is not the consequence of induced quiescence or apoptosis, thus the mechanisms of gene expression regulation by E2 only implicate its transcriptional regulatory properties, strongly influenced by its interaction with cellular proteins.

As expected, the results of the microarray analysis showed that HPV16 E2 affect a variety of cellular pathways (Tables 3 and 4), some of them altered in early stages of cervical cancer development, when E2 is still expressed before the integration of the viral genome into cell chromosome.

Interestingly we observed that the expression of a high number of genes on the WNT-pathway is modified for the expression of E2. In the last few years it has been reported that WNT-pathway is activated by the expression of E6 and E7 viral oncogenes [46-48]. However, our observations suggest that E2 is also targeting this pathway probably with different consequences than the induced by the viral oncogenes, since a tight regulation and controlled coordination of the WNT signaling cascade is required to maintain the balance between proliferation and differentiation. Recently it has been proposed that essentially all cellular information - i.e. from other signaling pathways, nutrient levels, etc. - is funneled down into a choice of coactivators usage, either CBP or p300, by their interacting partner beta-catenin (or catenin-like molecules in the absence of beta-catenin) to make the critical decision to either remain quiescent, or once entering cycle to proliferate without differentiation or to initiate the differentiation process [49]. Since CBP and p300 are also interactors for E2, the function of the WNT-pathway could be deeply modified by the low availability of the coactivators when the viral protein is present.

The control of this pathways in the viral cycle could have biological consequences as in the case of the regulation of cell proliferation, since the induction of Cyclin A expression by E2, orchestrated with a negative regulation of RhoA, known inhibitor of the cell proliferation, would allow the entry into the S phase of cell cycle [50-52]. Similarly, E2 expression could be also involved in apoptosis regulation since it negatively regulate genes involved in this process, such as caspase 9 (CASP9) [53], whose product is an effector of cell death. In the same way, EGR2 [54,55] is negatively regulated bringing as a consequence the inhibition of cytochrome c releasing it from the mitochondria. Interestingly, several genes mainly expressed in keratinocytes from the basal layers of stratified epithelia, such as type I keratins (keratin 14, 24 and 34) [56-58], were down-regulated in cells expressing E2 suggesting that the process of cell differentiation could be also regulated by this viral product.

## Conclusion

In conclusion, our results in this work demonstrate that HPV16 E2 has a regulatory effect on cellular gene expression independently of the viral oncoproteins E6 and E7. The analysis data presented in this study demonstrates that E2 predominantly induces a down-regulation of gene expression. The gene profile observed in E2 expressing cells suggests that E2 could induce these changes by its interactions with ubiquitous cellular proteins such as Sp1. Several genes involved in pathways altered in early stages of cervical cancer, such as CASP9 and EGR2 involved in apoptosis and MYC-N, CCNA and RhoA involved in cell proliferation, were altered by HPV16 E2 expression. The cellular processes affected suggest that E2 expression leads to the cells in to a convenient environment for a replicative cycle of the virus.

## Abbreviations

HPV: Human Papillomavirus; HR: High-Risk; CC: Cervical Cancer; TAF1: TBP-associated factor 1; PANTHER: Protein ANalysis Through Evolutionary Relationships; DMEM: Dulbecco's modified Eagle's medium; HEK293: Human Embryonic Kidney 293; FBS: Fetal Bovine Serum; DMEM-F12: Dulbecco's modified Eagle's medium: Nutrient Mixture F12; MLP: Major Late Promoter; HCMV: Human Cytomegalovirus; moi: multiplicity of infection; RT-qPCR: Quantitative Reverse Transcription PCR; ΔΔ Ct: Double delta Ct;

## Competing interests

The authors declare that they have no competing interests.

## Authors' contributions

ERS participated in the design of the study, carried out the microarray assays, microarray data analysis, data mining and real time PCR studies and participated in drafting manuscript. FC participated in real time PCR studies. AVH participated in the design of the adenoviral vectors and participated in drafting the manuscript. KN participated in the design of the adenoviral vectors. MS participated in the microarray data analysis. EG conceived the study participates in its design and coordination and revised the manuscript. All authors read and approved the final version of this manuscript.

## Supplementary Material

Additional file 1 **Up-regulated genes in C-33A cells by HPV16 E2 expression**. Z score values represent the change in gene expression related to non-infected cells. Fold Change refers to differences in Z score values compared to expression in non-infected cells. Analysis was performed using genArise software, changes in gene expression with a value ≥ 2.0 were considered as altered genes, the *p-*value was calculated by Student's t-test. Click here for file

Additional file 2 **Down-regulated genes in C-33A cells by HPV16 E2 expression**. Z score values represent the change in gene expression related to non-infected cells. Fold Change refers to differences in Z score values compared to expression in non-infected cells. Analysis was performed using genArise software, changes in gene expression with a value ≤ -2.0 were considered as altered genes, the *p-*value was calculated by Student's t-test. Click here for file
